# Distribution of SOCD along different offshore distances in China's fresh-water lake-Chaohu under different habitats

**DOI:** 10.1038/s41598-022-18260-2

**Published:** 2022-08-29

**Authors:** Xiaojie Yao, Jingjing Wang, Xinyun Xie, Dan Jiang, Xiaoniu Xu

**Affiliations:** 1 School of Forestry and Landscape Architecture, Anhui Agricultural University, Hefei, 230036 China; 2 College of Architecture and Urban Planning, Anhui Jianzhu University, Hefei, 230601 China; 3 Anhui Academy of Forestry, Hefei, 230031 China

**Keywords:** Forest ecology, Riparian ecology, Biogeochemistry, Environmental sciences

## Abstract

Carbon storage in wetland ecosystems is an important part of the carbon cycle of terrestrial ecosystems and provides important ecosystem services. Chaohu Wetland is a typical freshwater lake wetland in China. In this study, soil and plant samples were collected every 500 m through three sample lines of different vegetation habitats (estuarine banks, woodlands and shrub beaches) and different offshore distances, revealing the spatial distribution characteristics of soil organic carbon density (SOCD) in Chaohu wetland. The overall SOCD of Chaohu wetland was low, with different habitats ranking as Woodland > Estuary and riverside > Shrub and beach. SOCD of different offshore distances had no obvious law, and the SOCD decreased significantly with soil depth. The plant biomass was significantly higher at the woodland habitat than at other habitats. Most of soil nutrient indicators were the highest at the woodland habitat, while the estuary-riverside habitat had the highest N and P contents. Soil and plant nutrients at different offshore distances had no obvious change patterns. The contents of soil K, Ca, Mg, and N were significantly positively correlated with SOCD, but soil bulk density and pH were significantly negatively correlated with SOCD, and vegetation P content was significantly negatively correlated with SOCD. The spatial pattern of SOCD changes in this lake coastal wetland was determined by the combined effects of plant nutrients, biomass, and soil physical and chemical properties. Our results indicate Chaohu wetlands may have been experiencing serious degradation. The SOCD of Chaohu wetland is lower than that of other wetlands in China, which is mainly affected by human activities. Different offshore distances and habitat heterogeneity are the main factors affecting the soil carbon cycle of the wetland.

## Introduction

Wetlands account for only 5–8% of Earth’s terrestrial area, but they store about 30% of the carbon (C) pool of the global terrestrial ecosystem^1^. Because of the huge reserves of organic C, small changes of C pool in wetlands can greatly affect the atmospheric CO_2_ concentration^2^. Therefore, dynamic changes in wetland C storage have a significant impact on the global C cycle and climate change^3,4^. In addition, the increasing global warming will contribute to the sink-source transformation due to increased soil organic carbon (SOC) decomposition. Therefore, both reducing emissions and increasing the C sequestrations in the ecosystems are the important measures to alleviate excessive atmospheric CO_2_ concentrations^5^. Having strong C accumulation capability and high SOC storage^5,6^, wetlands have been paid more and more attention for the potential to mitigate climate change^7^.

Wetlands are the most ecological valuable ecosystems in the world, providing carbon sink^4,5^, biodiversity conservation, water purification, flood mitigation, coastal protection, and erosion control^8–10^. However, due to human disturbances, approximately half of wetlands in the world have been lost or degraded^11^. The major threats to wetlands are agricultural cultivation and urbanization, which have significantly reduced their SOC stocks and even resulted in loss of their ecological functioning^12,13^. Therefore, it is necessary to call for immediate attention to the restoration of wetland ecosystems^8,12^.

Soil C sequestration is one of the important functions of wetlands. Ecological restoration of wetlands has been conducted worldwide, which is considered as an effective measure to regain SOC lost as a result of human disturbances^4,7,14,15^. In the past few decades, great efforts were made in evaluation of soil C storages and their C sequestration potentials in the different wetland ecosystems^4,7,14^. The spatial distribution pattern of wetland SOC is influenced by a great number of factors, such as soil properties, climate, vegetation, hydrology, and land use patterns^7,16,17^. These factors and their interactions are extremely complex in wetlands ecosystems^18^. Therefore, there is a remarkable lack of understanding regarding the most influential factors determining SOC changes across various conditions. It is essential to conduct further studies on spatiotemporal patterns of SOC storage for different types of wetland ecosystems.

Lake Chaohu, located in the lower reach of the Yangtze River, is one of the five largest freshwater lakes in China. It is a shallow, eutrophic lake, with a surface area of^19^ 780 km^2^, The ecological restoration and protection of Chaohu coastal wetland have been the important research issues for the past dozen years^20–22^. However, the field investigation data of SOC for the coastal wetland of Lake Chaohu are very limited^23^. This results in a remarkable lack of understanding regarding the most influential factors controlling SOC changes across various conditions. Such an understanding is especially important for managing restored wetlands for the purpose of SOC recovery. In this study, our objectives are to (1) reveal the spatial distribution pattern of SOC density, (2) determine the key influential factors controlling SOC changes, and (3) provide theoretical support for managing and utilizing wetlands with the aim of increasing soil C sequestration.

## Materials and methods

### Study area description

Lake Chaohu (117° 16′ 54″–117° 51′ 46″ E, 30° 25′ 28″–31° 43′ 28″ N) is located to the north of the lower Yangtze River, with a drainage area of 9258 km^2^ and a replenishment coefficient of 12. The replenishment water makes up 98% of the total runoff into the lake, while precipitation over the lake only accounts for 2%. The entire basin is covered by 33 rivers, 760 km^2^ of lake area, and 28.56 km^2^ of beach area. This region is located in the northern subtropical zone and is characterized by a monsoon-influenced humid subtropical climate. The average annual precipitation is 1000–1158 mm. The average annual temperature is 15.9 °C. Soils in the region are primarily derived from river and lake sediment and mountain river alluvium and consist of largely paddy soil and fluvo-aquic soil in the lowlands, calcareous soil, yellow–brown soil and purple soil in the uplands.

### Sample collection

**Figure 1:**
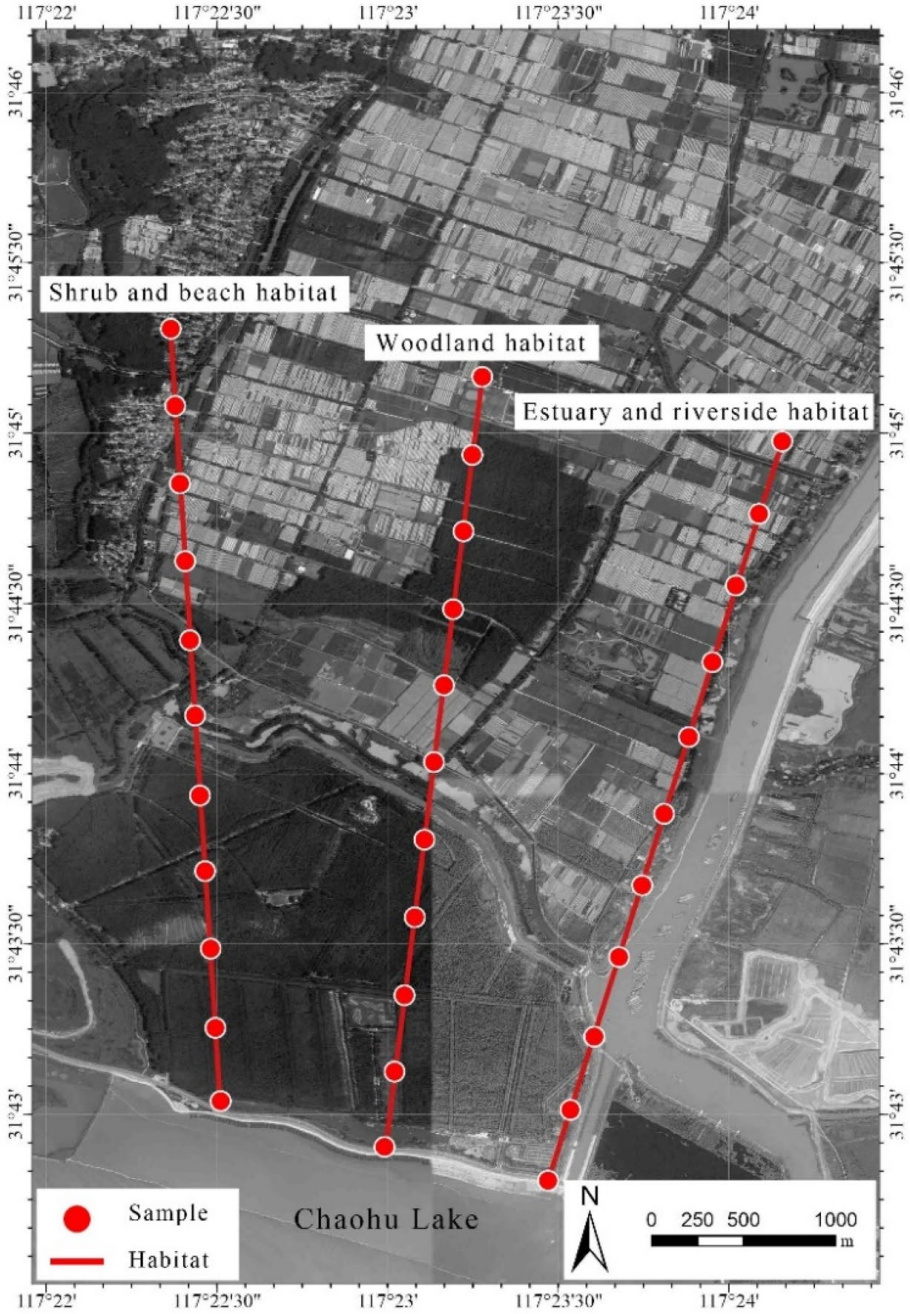
Location and distribution of sampling points in the Lake Chaohu wetland. Figure created with WeMap Version 3.9.1. http://www.rivermap.cn/index.html.

According to the vegetation types around Lake Chaohu, a total of 3 transects (estuary-riverside habitat, woodland habitat, and irrigation beach habitat) are set up perpendicular to the Lake Chaohu shoreline, with a distance of 2500 m between the transects, as shown in Fig. 1. According to the distribution of plants and different water level gradients, 11 plots with a total length of 5000 m are set up for each sample line at 500 m intervals. There are 3 samples of 1 m × 1 m in each plot, a total of 99 samples. Plant samples are collected from all the above-ground parts of the herbaceous plants in the sample frame, mixed and weighed to calculate the vegetation biomass, and part of the plant samples are taken to determine plant nutrients. The soil samples of 0–20 cm and 20–40 cm soil layers were collected respectively, and the samples were mixed at 3 points to determine the physical and chemical properties of the soil.

### Plant and soil sample measurements

The collected soil samples were air-dried, crushed, and sifted with a 2 mm sieve. Thoroughly mixed samples were sealed in sample bags for future use. The plant samples were dried at 70 °C to constant weight, crushed, and sifted with a 100-mesh sieve. The samples were stored in sealable sample bags. To measure soil pH (H_2_O), distilled water and soil samples were mixed in a 2.5:1 ratio (volume : mass), shaken well, and left undisturbed for 30 min. Measurement was then performed with a pH meter. To measure soil conductivity, distilled water and soil samples were mixed in a 5:1 ratio (volume : mass), shaken well, and left undisturbed for 1 h. Measurement was then performed with an Extech II conductivity meter and a pH meter. The organic carbon and total N in plant and soil samples were quantified by combustion using an EA3000 elemental analyzer (Vector, Italy). The total P in plants and soil was determined by nitric acid-perchloric acid digestion and molybdenum-antimony anti-spectrophotometry. Available P was extracted with hydrochloric acid-ammonium fluoride extractant, whereas ammonium nitrogen and nitrate nitrogen were extracted with 2 mol·L^−1^ KCl solution, all of which were measured using a FIA Star 5000 flow injection analyzer (FOSS, Denmark). Soil dissolved organic carbon (DOC) and total dissolved nitrogen (DN) were extracted with 2 mol·L^−1^ K_2_SO_4_ solution, determined by a Multi 3100 C/N analyzer (Jena, Germany).The soil bulk density is determined by the ring knife method. K, Na, Ca, and Mg in soil and plant samples were extracted by nitric acid-perchloric acid digestion and measured by atomic absorption spectroscopy (TAS-990 AFG, Beijing Persee General Analytical Instruments, China)^24^.

### Data analysis

SigmaPlot 14.0 was used to analyze the frequency distribution of SOC density in Lake Chaohu wetlands and to plot the histograms. Multi-factor analysis of variance and multiple comparisons were performed with SPSS 25.0 to find the degree of influence of habitat, offshore distance, and soil depth on SOCD. Linear regression models of SOCD vs. each variable (plant nutrients and soil physicochemical properties) were established using scatter plots and correlation analysis. Furthermore, a structural equation model was established based on soil physicochemical properties, plant nutrients, and plant biomass indicators, and the conversion factors that significantly affected SOCD were screened^25^.

The calculation formula of SOCD is as follows^26^:

1 $${SOCD}_{i}={C}_{i}{\times P}_{i}{\times H}_{i}\times {10}^{-2}$$

In the formula, *SOCD*_*i*_ is the soil organic carbon density of the *i*th layer (kg/m^2^); *C*_*i*_ is the soil organic carbon content of the *i*th layer (g/kg); *P*_*i*_ is the soil bulk density of the *i*th layer (g/cm^3^); *H*_*i*_ is the profile depth (cm); 10^–2^ is the unit conversion factor.

## Results

### Content characteristics of SOCD, soil physicochemical properties, plant nutrients and biomass

**Figure 2:**
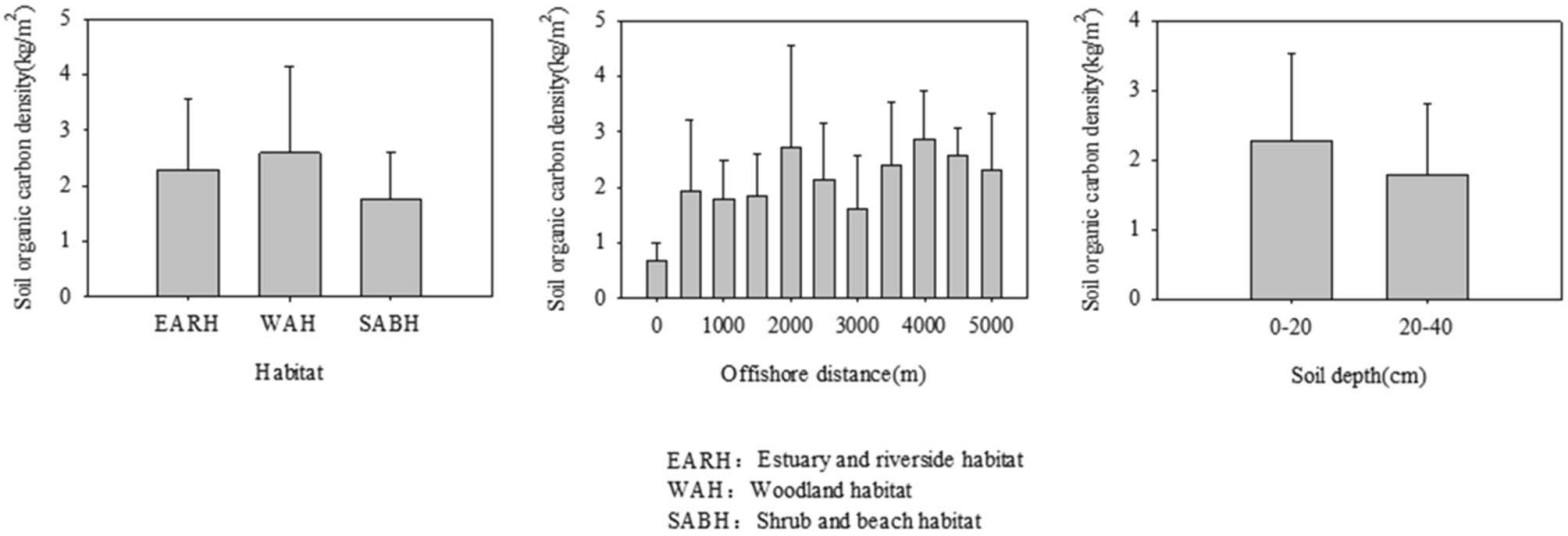
SOCD content of different types of soil.

As shown by the multi-factor analysis of variance (Table 1), all of habitat (*p* = 0.005), offshore distance (*p* = 0.002) and soil depth (*p* = 0.027) had significant influence on soil organic carbon in wetlands. The estuary-riverside habitat had the lowest average SOCD (2.29 kg/m^2^), while the woodland habitat had the highest (2.59 kg/m^2^). Comparing the SOCD at different offshore distances, the average SOCD at 0 m offshore was the lowest (0.70 kg/m^2^), and it was the highest at 4000 m offshore (2.88 kg/m^2^). The average SOCD of soil depths at 0–20 cm (2.28 kg/m^2^) significantly higher than 20–40 cm (1.80 kg/m^2^) (Fig. 2).

**Table 1:** Between-subjects effects of different variables on soil organic carbon density (SOCD, kg·m^−2^).

Source	Degree of freedom	F	Significance
**Dependent variable: soil organic carbon density (kg·m**^**−2**^)			
Modified model	13	3.830	< 0.001
Intercept	1	407.891	< 0.001
Habitat	2	5.666	0.005
Offshore distance	10	5.053	0.002
Soil depth	1	5.053	0.027
Error	91		
Total	105		
Total after correction	104		

R^2^ = 0.354 (Adj R^2^ = 0.261).

In different habitats, the soil nutrient content in woodland habitat was the highest, and there was no obvious rule in the change of soil nutrient content at different offshore distances (Table 2). The plant biomass and carbon content in woodland habitat were the highest, the N and P content in estuary-riverside habitat were the highest, and the change of offshore distance of plant nutrients was not obvious (Table 3).

**Table 2:** Variation characteristics of soil nutrients.

Different conditions	Indicators of soil nutrients								
	Bulk density (g·cm^−3^)	P (g·kg^−1)^	K (g·kg^−1^)	Ca (g·kg^−1^)	Mg (g·kg^−1^)	N (g·kg^−1^)	C/N	C/P	N/P
**Habitat conditions**									
Shrub-beach	1.02	0.12	3.55	1.13	3.58	1.17	9.38	157.52	15.95
Woodland	1.00	0.20	5.69	2.07	4.50	1.90	7.56	73.20	10.58
Estuary-riverside	0.85	0.17	5.15	1.12	2.39	0.93	13.15	74.13	6.26
**Offshore distance**									
0	1.04	0.16	2.32	1.66	2.65	0.22	18.85	22.76	1.45
500	0.95	0.13	3.74	0.93	2.24	0.90	12.63	76.68	6.63
1000	0.96	0.19	5.03	1.19	3.34	1.04	10.39	52.65	6.12
1500	0.99	0.14	4.30	1.12	2.76	1.04	9.74	70.36	7.80
2000	0.90	0.15	4.63	1.97	3.21	1.27	10.90	115.57	11.34
2500	0.88	0.12	5.71	0.90	3.39	1.37	9.75	120.89	12.63
3000	0.85	0.17	5.03	0.93	3.00	1.18	9.02	59.44	7.23
3500	1.00	0.17	6.01	1.73	3.51	1.28	11.05	86.64	8.89
4000	0.83	0.13	4.09	1.63	3.01	1.78	9.98	211.88	20.66
4500	0.84	0.22	6.76	1.21	3.21	1.58	10.51	108.27	10.67
5000	0.84	0.25	6.34	1.34	3.40	1.50	10.29	112.97	10.59

**Table 3:** Variation characteristics of plant nutrients and community biomass.

Different conditions	Indicators of plant nutrients and community biomass						
	P (g·kg^−1^)	C (g·kg^−1^)	N (g·kg^−1^)	C/N	C/P	N/P	Biomass (kg·hm^−2^)
**Habitat conditions**							
Shrub-beach	0.98	408.88	14.39	33.24	562.07	15.21	3503.40
Woodland	0.80	413.72	11.24	43.06	761.12	19.08	12,892.73
Estuary-riverside	1.42	395.69	17.73	29.83	862.21	29.95	2421.73
**Offshore distance**							
0	2.56	361.36	18.55	24.62	256.93	8.72	3089.78
500	1.53	404.08	15.40	34.37	564.31	12.90	4492.22
1000	2.85	413.23	24.13	21.25	211.67	10.51	2690.33
1500	1.73	396.49	16.37	33.79	343.25	10.03	6598.56
2000	1.09	418.95	13.60	39.65	629.38	18.19	8199.33
2500	1.24	421.79	16.85	31.70	507.07	15.28	6857.34
3000	1.57	388.22	18.35	27.35	385.98	14.78	8603.22
3500	0.53	387.74	14.61	35.57	1989.73	64.65	6922.22
4000	0.46	424.15	16.82	30.89	1293.52	48.61	5552.33
4500	0.58	392.96	9.54	45.61	880.34	20.85	7022.50
5000	0.67	406.56	15.83	32.72	977.42	36.06	10,144.00

### Correlation among SOCD, soil physicochemical properties, plant nutrients and biomass

**Figure 3:**
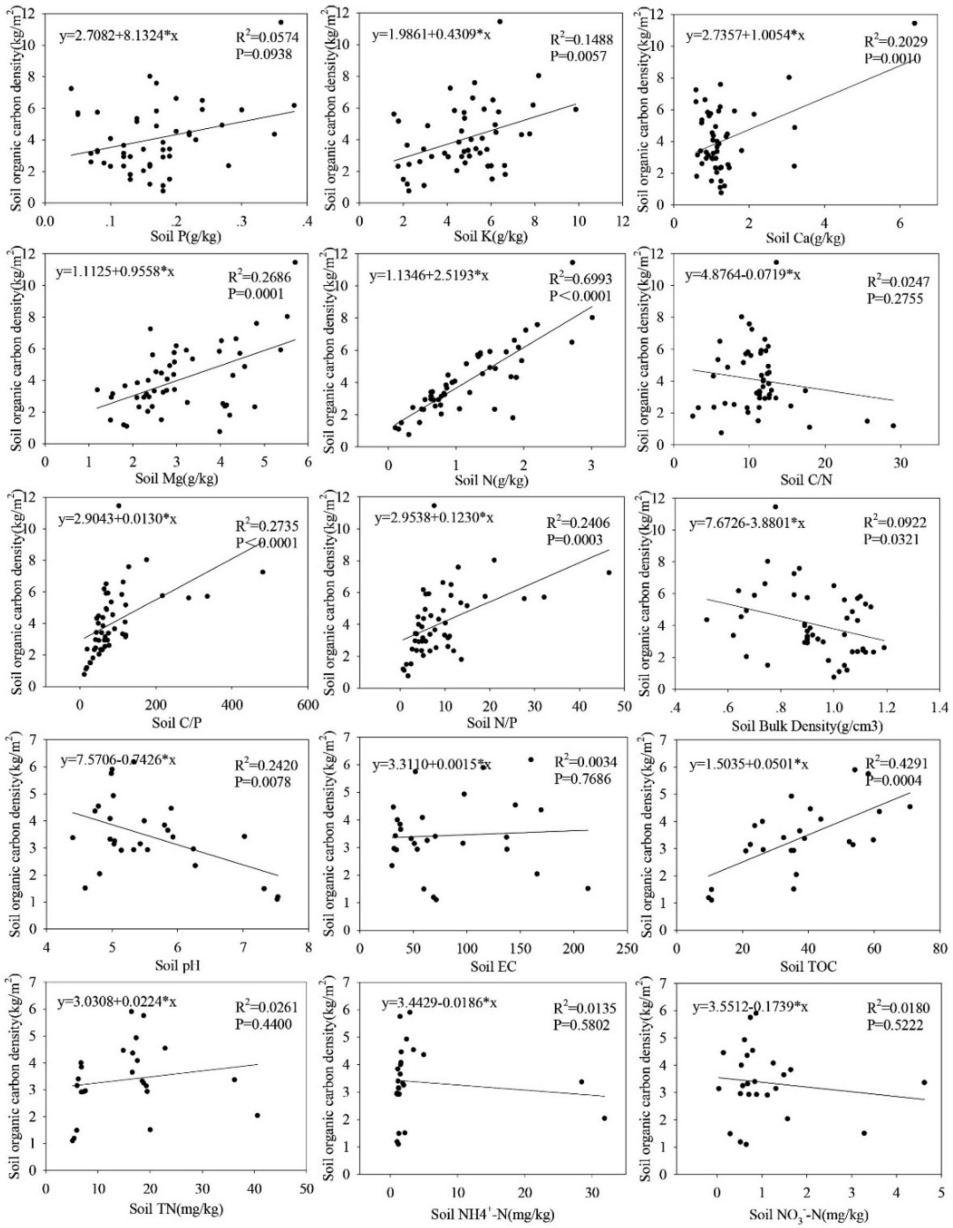
Determine the soil organic carbon density (SOCD, kg·m^−2^) and soil nutrient factor content under each sample, and correlate between soil organic carbon density (SOCD, kg·m^−2^) and soil factors (mg·kg^−1^) (including Soil P, K, Ca, Mg, N, C/N, C/P, N/P, Bulk Density, pH, EC, TOC, TN, NH4^+^–N and NO3^−^–N) in the same space.

**Figure 4:**
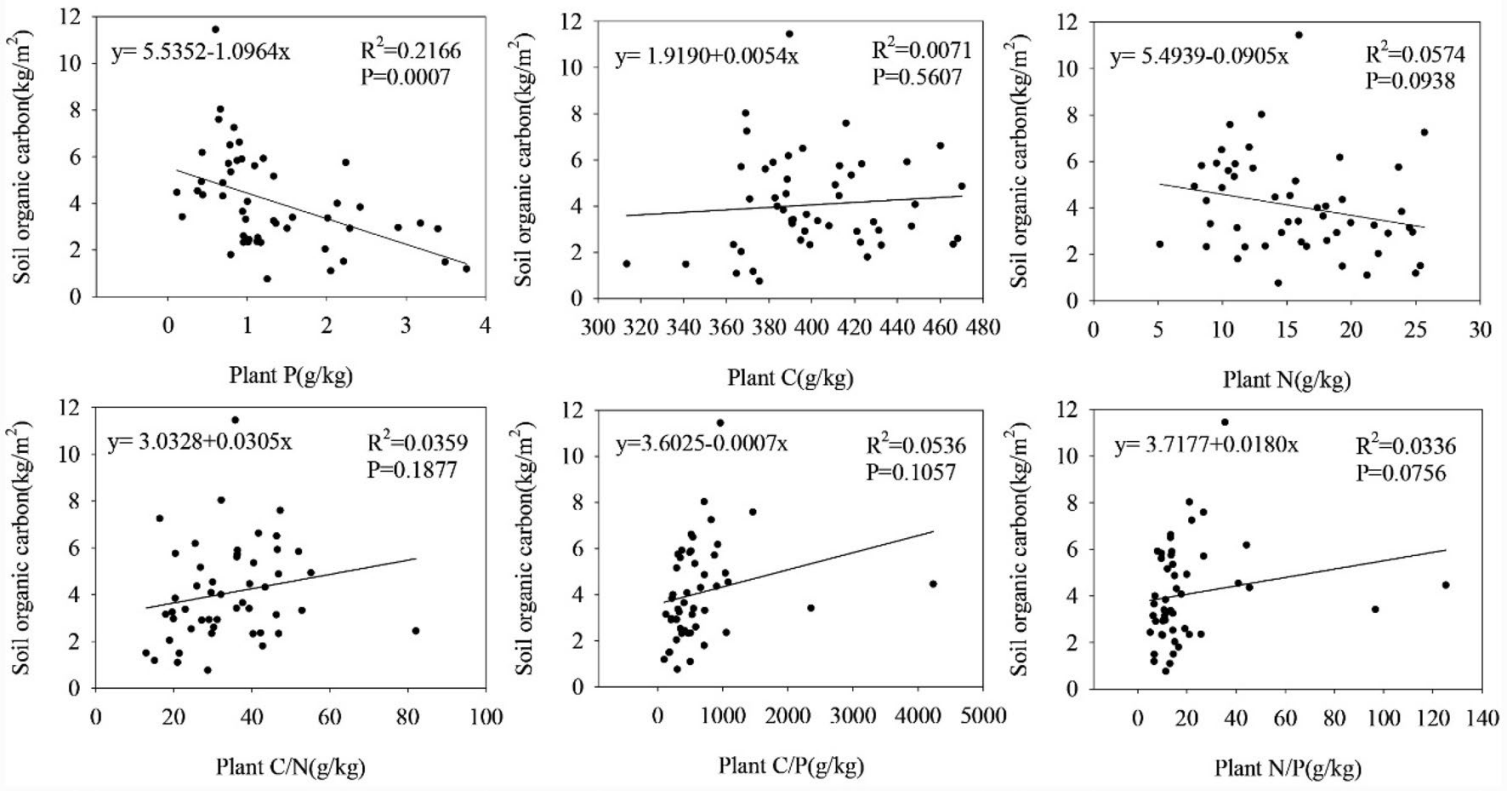
Determine the soil organic carbon density (SOCD, kg·m^−2^) and plant nutrient factor content under each sample, and correlate the soil organic carbon density (SOCD, kg·m^−2^) with Plant P (g·kg^−1^), Plant C, Plant N, Plant C/N, Plant C/P, and Plant N/P in the same space.

As shown by Fig. 3, contents of K (*p* = 0.0057), Ca (*p* = 0.001), Mg (*p* = 0.0001), N (*p* < 0.0001), C/P (*p* < 0.0001), N/P (*p* = 0.0003) and TOC (*p* = 0.0004) in soil were significantly positively correlated with SOCD while soil bulk density (*p* = 0.0321) and pH (*p* = 0.0078) showed significant negative correlation with SOCD (*p* < 0.05). Among the plant nutrients, only P (*p* = 0.0007) showed a significant negative correlation with SOCD (Fig. 4).

### Controls of wetland SOCD pattern

**Figure 5:**
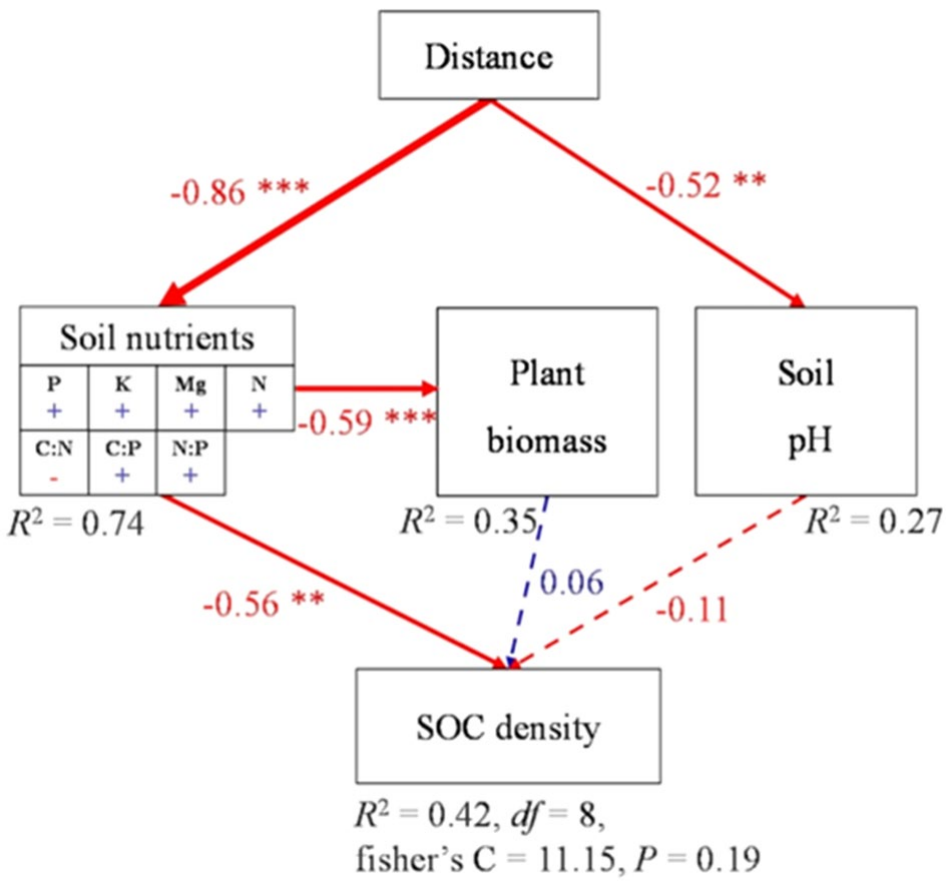
The construction of the best structure equation model (SEM), reveals the influence of various factors on soil organic carbon density (SOCD, kg·m^−2^). It is concluded that offshore distance further affects the change of SOCD through the inhibition of other factors (soil factor, biomass and soil pH).

The best structure equation model (SEM) explained 42% of the variations in SOCD spatial heterogeneity (Fig. 5). Vegetation biomass (standardized effect size: 0.06), but not plant nutrient parameters positively contributed to SOCD spatial heterogeneity. However, soil nutrient parameters (standardized effect size: − 0.56; p < 0.01) and other parameters (pH) (standardized effect size: − 0.11) negatively contributed to SOCD spatial heterogeneity. The offshore distance was significantly negatively related to vegetation biomass (standardized effect size: − 0.86; p < 0.001), and soil other parameters (standardized effect size: − 0.52; p < 0.01).

## Discussion

The distribution of soil organic carbon in the coastal wetlands of Lake Chaohu showed a significantly vertical decrease with soil depth, which was consistent with the previous studies^27,28^. The vertical distribution of SOC is dominantly affected by the primary productivity of plant community, litter yield and decomposition rate^29^. The source of SOC is mainly from belowground root turnover and aboveground litter input of vegetation, which mainly exist in the topsoil. This makes the SOC have characteristics of surface aggregation^30,31^.

Our results showed significant differences in SOCDs and SOC contents among the different habitats. This is due to the differences in plant community structures with different habits and photosynthetic fixation capacity, resulting in different quantity and quality of litter that has different effects on the carbon sink/source function of wetland soil^32^. The distinct differences in growth form, amount of aerenchyma, rooting depth, or timing and magnitude of primary production of different vegetation types have substantial influences on the spatial heterogeneity of SOCD in wetlands^33^. The spatial distribution of SOCD and accumulation of SOC in wetland ecosystems is a complicated process and is controlled by multiple factors, of which vegetation is regarded as one of the key factors^33,34^. In addition, the hydrologic regime has been considered as the driving force in C cycling in wetland ecosystems, which directly changes the wetland physicochemical properties, especially oxygen availability that controls decomposition of organic matter^35,36^. Furthermore, multiple environmental variables including soil properties, climate, and terrain have important impacts on the spatial distribution of SOC^35,36^. Diversity of micro-topography in natural wetland systems also plays an important role in affecting spatial distribution of SOC^33,36^.

Based on the optimal structural model, the offshore distance was a key factor influencing SOCD at our site. It could be mainly caused by the change of soil nutrient, pH, and vegetation biomass. Factually, to a certain extent, offshore distance reflects the changes of hydrological regime. As water-controlled ecosystems, wetland vegetations respond to the water level fluctuation. It has been well illustrated a close relationship between SOC and altitudinal gradient^36,37^. Zhao et al. reported that pH has a significant correlation with the SOC content^35^. It is in agreement to our results.

The average SOCD of the wetland in Chaohu is much lower than that of other wetlands in China (16.8 kg·m^−2^)^38^. Soil organic carbon pool is a dynamic equilibrium process and varies depending on input and output differences of carbon sources^39^. Due to the high sensitivity of wetland soil carbon to the changes of the surrounding environment^40^, Chaohu is a densely populated area with a rapidly developing located in the administrative territorial entity economy, this is an important reason for the decrease of wetland soil carbon storage. Human disturbances seriously affect the carbon sequestration capacity of Chaohu 's ecosystems. Therefore, balancing the economic and ecological relationship is important for stabilizing the carbon cycle in the region around Lake Chaohu.

## Conclusions

The SOCD in coastal wetland of Lake Chaohu was significantly higher in topsoil than that in subsoil, which is mainly related to the distribution of litter and root system. SOCD was higher in woodland habitat than in the others. SOCD within 500-m offshore distance was lower than 500 m away. With the increase of offshore distance, SOCD increased nonlinearly that was related to soil pH, vegetation biomass and soil nutrients. There existed great spatial heterogeneity of soil organic carbon distributions in wetland of Lake Chaohu. However, its internal driving mechanism is not entirely clear. Therefore further researches are needed on the factors affecting SOC distribution in this coastal wetlands.

## Data Availability

The data that support the findings of this study are available from the corresponding author upon reasonable request.

## References

[1] Mitsch WJ (2013). Wetlands, carbon, and climate change. Landsc. Ecol..

[2] Koehler AK, Sottocornola M, Kiely G (2015). How strong is the current carbon sequestration of an Atlantic blanket bog?. Glob. Change Biol..

[3] Chmura GL, Anisfeld SC, Cahoon DR, Lynch JC (2003). Global carbon sequestration in tidal, saline wetland soils. Global Biogeochem. Cycles.

[4] Dong HY, Qian LW, Yan JF, Wang L (2020). Evaluation of the carbon accumulation capability and carbon storage of different types of wetlands in the Nanhui tidal flat of the Yangtze River estuary. Environ. Monit. Assess..

[5] Zaher H, Sabir M, Benjelloun H, Paul-Igor H (2020). Effect of forest land use change on carbohydrates, physical soil quality and carbon stocks in Moroccan cedar area. J. Environ. Manage..

[6] Friborg T (2003). Siberian wetlands: Where a sink is a source. Geophys. Res. Lett..

[7] Dayathilake D, Lokupitiya E, Wijeratne V (2021). Estimation of soil carbon stocks of urban freshwater wetlands in the Colombo Ramsar Wetland City and their potential role in climate change mitigation. Wetlands..

[8] Li XW (2014). How important are the wetlands in the middle-lower Yangtze River region: An ecosystem service valuation approach. Ecosyst. Serv..

[9] Liu K (2014). Diversity of vascular plant and classification system of vegetation in wetlands of Anhui Province. Acta Ecol. Sin..

[10] Liu H, Zheng L, Wu J, Liao YH (2020). Past and future ecosystem service trade-offs in Poyang Lake Basin under different land use policy scenarios. Arab. J. Geosci..

[11] Dixon MJR (2016). Tracking global change in ecosystem area: the Wetland Extent Trends index. Biol. Conserv..

[12] Yang X, Liu S, Jia C, Liu Y, Yu CC (2021). Vulnerability assessment and management planning for the ecological environment in urban wetlands. J. Environ. Manag..

[13] Ghosh S, Das A (2019). Urban expansion induced vulnerability assessment of East Kolkata Wetland using Fuzzy MCDM method. Remote Sens. Appl. Soc. Environ..

[14] Means MM, Ahn C, Korol AR, Williams LD (2016). Carbon storage potential by four macrophytes as affected by planting diversity in a created wetland. J. Environ. Manag..

[15] Fenstermacher DE, Rabenhorst MC, Lang MW, McCarty GW, Needelman BA (2016). Carbon in natural, cultivated, and restored depressional wetlands in the Mid-Atlantic Coastal Plain. J. Environ. Qual..

[16] Abegaz A, Winowiecki LA, Vågen T, Langan S, Smith JU (2016). Spatial and temporal dynamics of soil organic carbon in landscapes of the upper Blue Nile Basin of the Ethiopian Highlands. Agric. Ecosyst. Environ..

[17] Xie E, Zhang Y, Huang B, Zhao Y, Qu M (2021). Spatiotemporal variations in soil organic carbon and their drivers in southeastern China during 1981–2011. Soil Tillage Res..

[18] Jackson RB (2017). The ecology of soil carbon: Pools, vulnerabilities, and biotic and abiotic controls. Annu. Rev. Ecol. Evol. Syst..

[19] Sun KK, Chen X, Dong XH, Yang XD (2020). Spatiotemporal patterns of carbon sequestration in a large shallow lake, Lake Chaohu: Evidence from multiple-core records. Limnologica.

[20] Chen X, Yang XD, Dong XH, Liu EF (2013). Environmental changes in Lake Chaohu (southeast, China) since the mid 20th century: The interactive impacts of nutrients, hydrology and climate. Limnologica..

[21] Yu JH (2021). Temporal changes in fractions and loading of sediment nitrogen during the holistic growth period of Phragmites australis in littoral Lake Chaohu, China. J. Lake Sci..

[22] Zhang M, Kong FX (2015). The process, spatial and temporal distrbition and mitigation strategies of the eutrophication of Lake Chaohu (1984–2013). J. Lake Sci..

[23] Teng Z, Cao XQ, Sun MY, Li PX, Xu XN (2019). Effect of different ecological restoration patterns on soil labile organic carbon and carbon pool management index of lakeside wetland of Lake Chaohu. Ecol. Environ. Sci..

[24] Wang JJ (2019). Effects of simulated nitrogen deposition on soil microbial biomass and community function in subtropical evergreen broad-leaved forest. For. Syst..

[25] Yang Y (2008). Storage, patterns and controls of soil organic carbon in the Tibetan grasslands. Glob. Change Biol..

[26] Li J (2020). The spatial distribution of soil organic carbon density and carbon storage in Baiyangdian wetland. Acta Ecologica Sinica.

[27] Ma WW (2018). Variations of organic carbon storage in vegetation-soil systems during vegetation degradation in the Gahai wetland, China. Chin. J. Appl. Ecol..

[28] Donato DC (2015). Mangroves among the most carbon-rich forests in the tropics. Nat. Geosci..

[29] Cao L (2013). Deposition and burial of organic carbon in coastal salt marsh: Research progress. Chin. J. Appl. Ecol..

[30] Liao XJ (2013). Distribution pattern of soil organic carbon contents in the coastal wetlands in Eastern Fujian. Wetl. Sci..

[31] Kong FL, Min XI, Yue LI, Li-Hua XU, Feng XM (2013). Distribution and storage of DOC in a typical annular wetland of Sanjiang Plain. Bull. Soil Water Conserv..

[32] He LP, Meng GT, Li GX, Li PR, Chai Y (2016). Soil organic carbon and its distribution characteristics in the soil profile under different vegetation recovery modes in toutang small watershed of Jinsha river. Resour. Environ. Yangtze Basin.

[33] Bernal B, Mitsch WJ (2008). A comparison of soil carbon pools and profiles in wetlands in Costa Rica and Ohio. Ecol. Eng..

[34] Dong J (2021). A novel organic carbon accumulation mechanism in croplands in the Yellow River Delta, China. Sci. Total Environ..

[35] Wang S, Adhikari K, Wang Q, Jin X, Li H (2018). Role of environmental variables in the spatial distribution of soil carbon (C), nitrogen (N), and C:N ratio from the northeastern coastal agroecosystems in China. Ecol. Indic..

[36] Zhao Q (2020). Soil organic carbon content and stock in wetlands with different hydrologic conditions in the Yellow River Delta, China. Ecohydrol. Hydrobiol..

[37] Weishampel P, Kolka R, King JY (2009). Carbon pools and productivity in a 1-km^2^ heterogeneous forest and peatland mosaic in Minnesota, USA. For. Ecol. Manag..

[38] Yu DS, Shi XZ, Wang HJ, Sun WX, Zhao YC (2007). Regional patterns of soil organic carbon stocks in China. J. Environ. Manag..

[39] Wu Y (2020). Elevation gradient characteristics and impact factors of soil carbon fractions in the *Pinus taiwanensis* Hayata forests of Daiyun Mountain. Acta Ecol. Sinica..

[40] Lal R (2004). Soil carbon sequestration impacts on global climate change and food security. Science.

